# Advances with RNAi-Based Therapy for Hepatitis B Virus Infection

**DOI:** 10.3390/v12080851

**Published:** 2020-08-04

**Authors:** Fiona van den Berg, Shonisani Wendy Limani, Njabulo Mnyandu, Mohube Betty Maepa, Abdullah Ely, Patrick Arbuthnot

**Affiliations:** Wits/SAMRC Antiviral Gene Therapy Research Unit, School of Pathology, Faculty of Health Sciences, University of the Witwatersrand, Johannesburg 2050, South Africa; Fiona.vandenBerg@wits.ac.za (F.v.d.B.); wendylimani@gmail.com (S.W.L.); nz.mnyandu@gmail.com (N.M.); Betty.Maepa@wits.ac.za (M.B.M.); Abdullah.Ely@wits.ac.za (A.E.)

**Keywords:** HBV, RNAi, siRNA, shRNA, miRNA, cccDNA

## Abstract

Infection with hepatitis B virus (HBV) remains a global health challenge. Approximately 292 million people worldwide are chronically infected with HBV and the annual mortality from the infection is approaching 900,000. Despite the availability of an effective prophylactic vaccine, millions of individuals are at risk of potentially fatal complicating cirrhosis and hepatocellular carcinoma. Current drug treatments can suppress viral replication, slow the progression of liver fibrosis, and reduce infectivity, but can rarely clear the viral covalently closed circular DNA (cccDNA) that is responsible for HBV persistence. Alternative therapeutic strategies, including those based on viral gene silencing by harnessing the RNA interference (RNAi) pathway, effectively suppress HBV replication and thus hold promise. RNAi-based silencing of certain viral genes may even lead to disabling of cccDNA during chronic infection. This review summarizes different RNAi activators that have been tested against HBV, the advances with vectors used to deliver artificial potentially therapeutic RNAi sequences to the liver, and the current status of preclinical and clinical investigation.

## 1. Introduction

Problems arising as a result of chronic infection with hepatitis B virus (HBV) continue to pose major global health challenges (reviewed in [1]). Annual mortality is estimated to be 880,000 and is now similar to the death rate caused by human immunodeficiency virus-1 (HIV-1). HBV infection is particularly common in sub-Saharan Africa, East and Southeast Asia, and the western Pacific islands, where complicating cirrhosis and hepatocellular carcinoma occur with high frequency. Although an effective prophylactic vaccine is available, it has little use for individuals who are already chronically infected with HBV. Moreover, available therapies have modest curative efficacy and are incapable of reliably eliminating all replication intermediates of the virus.

## 2. HBV Replication

**Figure 1:**
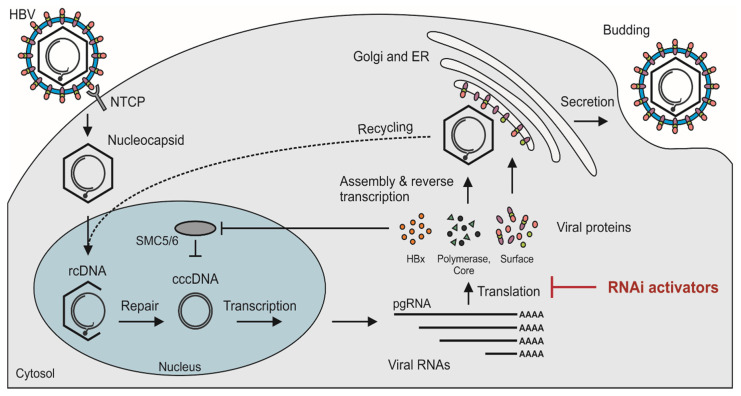
Disruption of the hepatitis B virus (HBV) replication cycle by RNA interference (RNAi) activators. HBV enters hepatocytes through a specific interaction with the sodium taurocholate co-transporting polypeptide (NTCP) receptor and the nucleocapsid is transported to the nucleus. Relaxed circular DNA (rcDNA) is then released and “repaired” to form covalently closed circular DNA (cccDNA). This stable intermediate is transcribed to produce viral RNAs, including pregenomic RNA (pgRNA), which are exported to the cytoplasm and translated. Encapsidation of the pgRNA, together with a viral polymerase, by the core proteins signals its conversion to rcDNA, thereby yielding a mature nucleocapsid. The nucleocapsids may then be recycled to replenish cccDNA in the nucleus (dashed line) or trafficked through the Golgi endoplasmic reticulum (ER), thereby acquiring surface antigen-embedded membranes, and then being secreted from the cell as a new infectious virions. The protein coded by the viral X open reading frame, HBx, targets the cellular SMC5/6 (structural maintenance of chromosomes) complex for degradation and thereby enables transcription from cccDNA. RNAi activators function to degrade target RNAs, thus preventing the translation of viral transcripts and inhibiting HBV replication. RNAi activators tested successfully against HBV include short interfering RNAs (siRNAs), short hairpin RNAs (shRNAs), and artificial primary microRNAs (pri-miRNAs).

The virion of HBV, or Dane particle, has large, middle, and small surface antigens (HBsAgs) embedded in a surrounding envelope (reviewed in [2]). The nucleocapsid is located within the envelope and comprises an icosahedral capsid, which is typically made up of 120 dimers of the core protein [3], with encapsulated viral relaxed circular DNA (rcDNA) and polymerase protein. In addition to the intact infectious particles, serum of HBV-infected individuals contains an abundance of subviral particles that are mostly made up of small surface protein [4,5,6]. Infection of hepatocytes is initiated by interaction of the Dane particle with glycosaminoglycans located on the cell surface [7]. Thereafter, specific binding of the myristylated large surface antigen to the sodium taurocholate polypeptide (NTCP) bile acid transporter initiates cellular entry of the nucleocapsid [8,9] (Figure 1). Evidence indicates that the epidermal growth factor receptor may also play a role in uptake of HBV into liver cells [10,11]. The elusive NTCP receptor of HBV was only discovered in 2012 and thereafter provided valuable impetus to research on HBV replication [8]. Prior to the discovery, work on HBV was hampered by a lack of information about how HBV gains access to hepatocytes during infection.

After traversing the cell membrane, the nucleocapsid is released and transferred to the nucleus [12] (Figure 1). This process is facilitated by nuclear localization signals of the core proteins. Regulated breakdown of capsids leads to release of rcDNA, which is then converted to covalently closed circular DNA (cccDNA) within the nucleus. The repair process involves use of host cell enzymes such as tyrosyl DNA phosphodiesterase 2 (TDP2) [13] and flap endonuclease 1 (Fen1) [14]. Conversion of rcDNA to cccDNA and establishment of HBV replication is an efficient process. Studies on chimpanzees [15] and ducks [16] have shown that a single infectious particle is sufficient to initiate infection with a replicating virus.

cccDNA is a stable replication intermediate and serves as the template for transcription of pregenomic RNA (pgRNA) and viral protein-encoding mRNAs [17]. Maintenance of cccDNA within infected hepatocytes is enabled by HBx, the protein encoded by the X open reading frame (ORF) [18]. Naturally, extrachromosomal DNA such as cccDNA is targeted for transcriptional silencing by the cellular structural maintenance of chromosomes 5/6 (SMC5/6) complex. HBx interacts with the damaged DNA-binding (DDB) domain of ubiquitin ligase 1 to render SMC5/6 unstable and thereby facilitate HBV gene expression [18] (Figure 1). ORFs of the cccDNA are arranged in a remarkably compact organization (reviewed in [2]). The protein-coding sequences overlap with each other and multiprotein coding is enabled by use of different reading frames. Further adding to the compact arrangement is the embedding of regulatory elements, such as promoters and enhancers, within the viral ORFs.

HBV transcripts are initiated at the basic core promoter/enhancer II, PreS1, S, and X promoters (reviewed in [19]). However, with only one poly(A) site on the cccDNA, the RNAs terminate at a common 3′ end. There are four viral ORFs: polymerase (P), core (C), surface (S), and X (reviewed in [20]). The polymerase protein is the largest viral protein and comprises 833 amino acids. This enzyme is responsible for priming, reverse transcription, and degradation of hybridized RNA (RNaseH action) during the synthesis of rcDNA from the pgRNA. The C ORF has two in-frame translation initiation sites: preC and C. Translation initiated at the preC AUG leads to expression of the preC/C fusion protein, which is processed in the endoplasmic reticulum and gives rise to the secreted HBV e antigen (HBeAg). This marker is a useful clinical indicator of active viral replication. Translation initiated at the C AUG gives rise to the capsid-forming core protein. The S ORF has three in-frame translation initiation codons: preS1, preS2, and S, which code for the large, middle, and small HBsAgs, respectively. Assembly of the viral particles is initiated by encapsidation of pgRNA and polymerase in the capsid particles. Reverse transcription of the pgRNA is commenced in the nucleocapsid. Subsequent secretion via the endoplasmic reticulum adds the envelope with surface proteins to release intact infectious virions. rcDNA-containing capsids may also be transported to the nucleus without secretion and thereby contribute to further replenishment of cccDNA. During replication, HBV per se causes minimal cytotoxicity. It is the inflammatory response to the infection that is largely responsible for hepatitis that accompanies HBV infection.

## 3. Goals of Treating Chronic Infection with HBV

Various types of cure from HBV infection have been defined [1]. A functional cure describes the status of a patient after HBsAg is eliminated, with or without seroconversion, but is usually associated with continued presence of cccDNA. Complete cure is a functional cure with elimination of cccDNA, and complete eradication refers to removal of all viral elements from an infected patient. Functional cure is currently thought to be a realistic goal of treatment strategies. However, complete sterilizing eradication of the virus, which would probably require combination therapy, is the ultimate goal of HBV therapy. A difficulty of assessing success of treatment is the lack of suitable biomarkers for measurement of intrahepatic cccDNA. Some recently described and potentially useful assays include evaluation of core-related HBV antigen (HBcrAg) [21] and serum viral RNAs [22]. These show promise, but thorough validation remains to be established. 

## 4. Currently Licensed Treatment for HBV

The rationale for treating infection with HBV is to minimize or eliminate complications that result from persistence of the virus. Currently there are two main classes of drug available to treat HBV infection: nucleoside/nucleotide analogues (NAs), which disrupt viral DNA synthesis during reverse transcription, and interferon-alpha (IFN-α) (reviewed in [23]). The six currently licensed NAs are lamivudine (LAM), adefovir dipivoxil (ADV), telbivudine (LdT), entecavir (ETV), tenofovir disoproxil fumarate (TDF), and tenofovir alafenamide (TAF). These drugs efficiently suppress viral replication and may inhibit formation of new cccDNA molecules, but they have little effect on established pools of cccDNA or formation of cccDNA in newly infected cells. The first-generation NAs—LAM, ADV, and LdT—are limited by their low barrier to viral resistance. TAF, TDF, and ETV are more effective and curb emergence of viral escape. IFN-α is usually administered as a pegylated molecule to improve bioavailability and durability of antiviral action. IFN-α has various antiviral actions and not all are completely understood. In addition to immunomodulation, IFN-α acts to inhibit viral DNA replication. Although a few cases of seroconversion from HBsAg positive to negative status have been reported in HBV-infected individuals treated with IFN-α (reviewed in [1]), the drug is not without drawbacks. It is expensive and also associated with side effects. Moreover IFN-α is contraindicated in HBV carriers with decompensated cirrhosis.

## 5. New HBV Drugs under Development

A variety of new approaches is being developed for treatment of chronic infection with HBV. Gene editing has gained popularity and is based on targeted mutation of HBV sequences, particularly cccDNA (reviewed in [24]). Use of transcription activator-like effector nucleases (TALENs) and RNA-guided endonucleases derived from the clustered regularly interspaced short palindromic repeats (CRISPR) and CRISPR-associated (Cas) system have shown good efficacy. The underlying rationale for the approach is based on achieving specific mutation of HBV sequences following repeated target cleavage and subsequent error-prone repair by non-homologous end joining (NHEJ). Evidence from preclinical studies has been promising. Evaluation of efficacy in a clinical setting will likely be undertaken when challenges of ensuring specificity and efficient delivery of sequences encoding gene editors to HBV-infected hepatocytes have been met. The capsids of HBV enhance formation of cccDNA by regulating nuclear import of nucleocapsids and encapsulation of pgRNA prior to reverse transcription. Capsids have therefore been the target of a new class of drugs termed capsid assembly modulators (CAMs) (reviewed in [25]). Several candidates have shown promise in preclinical evaluations and are being developed for clinical translation. Importance of the interaction between the myristoylated large HBsAg and the NTCP receptor has been exploited to develop HBV entry inhibitors [26,27,28]. MyrcludexB (Bulevirtide), a drug candidate comprising a truncated large HBsAg with N-terminal myristoyl that functions as a competitive inhibitor of viral entry is now in phase 3 clinical trial (clinicaltrials.gov, NCT03852719).

### 5.1. Rationale for Advancing RNAi-Based Anti-HBV Therapy

RNA molecules, both pgRNA and mRNAs encoding viral proteins, are essential for HBV replication. Destabilizing these viral sequences is therefore justifiably thought to be a good strategy for inactivating the virus. By effecting degradation of pgRNA, formation of cccDNA is countered. Moreover, inhibition of translation of viral proteins that are important for cccDNA formation, such as C and HBx, should also limit cccDNA production. Compact arrangement of the HBV genome limits sequence plasticity and ability to evade silencing sequences. Furthermore, presence of common 3′ sequences that include *HBx* means that different viral transcripts may be targeted by individual silencing sequences (Figure 1).

### 5.2. The RNAi Pathway

RNA interference (RNAi) is a ubiquitous post-transcriptional gene silencing (PTGS) mechanism found in metazoan cells. Potent and specific gene silencing is affected by small duplex RNAs such as 21–23 nt short interfering RNAs (siRNAs) and ≈22 nt microRNAs (miRNAs). In mammalian cells, miRNAs play a fundamental role in gene regulation and are generated by the miRNA biogenesis pathway (reviewed in [29]). Although the basic steps of this pathway are well known, additional regulatory factors and mechanisms have been recently described (reviewed in [30]).

Genes encoding miRNAs are transcribed to produce precursor transcripts from which the mature sequences are generated in a stepwise manner that involves action of RNase III enzymes (reviewed in [29]). Characteristic hairpin structures in the transcript constitute primary miRNAs (pri-miRNAs). Sequence motifs in the pri-miRNA are recognized for processing by the nuclear Drosha–DGCR8 microprocessor complex. The product is a shorter hairpin known as a precursor miRNA (pre-miRNA), which is exported from the nucleus by Exportin-5 in a Ran-GTP-dependent manner through nuclear pore complexes. In the cytoplasm, the terminal loop of the pre-miRNA is cleaved by the RNase III enzyme Dicer [31] to produce an imperfectly paired ≈22 bp miRNA duplex. The ends of the duplex have 2 nt 3′ overhangs as a result of RNase III cleavage by Drosha and Dicer.

One strand of the miRNA duplex is selected as the mature miRNA or guide sequence and loaded onto an Argonaute (Ago) protein of the RNA-induced silencing complex (RISC) (reviewed in [32]). Prior to loading, Dicer associates with the TAR (trans-activation response) RNA-binding protein (TRBP) to form the RISC loading complex (RLC). RISC loading is asymmetric as the strand of the miRNA duplex with the lower 5′ stability is selected as the mature miRNA. There are four closely related Ago proteins (Ago 1–4) that may associate with the miRNA, but only Ago2 has endonuclease activity [33,34].

The miRNA typically directs RISC to partially complementary binding sites in the 3′ untranslated region (UTR) of messenger RNAs (mRNAs), which are then targeted for translational repression, deadenylation, and degradation (reviewed in [35]). Alternatively, if there is near-perfect complementary base pairing between the miRNA and mRNA, the mRNA may be cleaved by an Ago2-RISC. This is often the case with artificial RNAi activators that have been developed for anti-HBV therapeutic application. These artificial sequences are designed to function as intermediates at different steps of the miRNA biogenesis pathway to reprogram silencing for therapy. As with natural intermediates, sequence and structural features of RNA activators are important to achieve efficient processing gene silencing.

### 5.3. RNAi Activators

**Figure 2:**
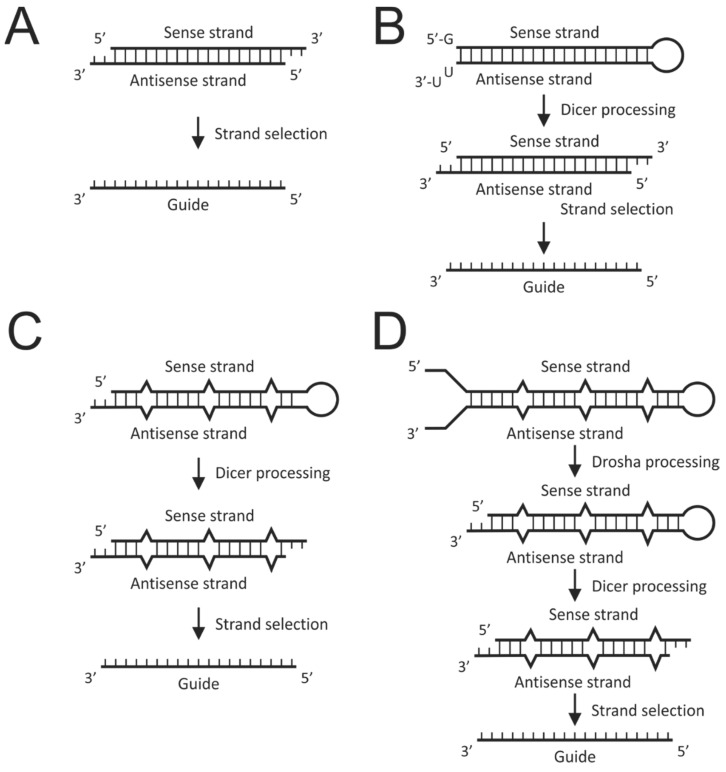
Synthetic and expressed activators of the RNA interference (RNAi) pathway. (**A**). Small interfering RNA (siRNA), typically produced as synthetic sequences, are perfectly matched 19–21 nucleotide duplex RNAs with two nucleotide 3′ overhangs. Each strand contains terminal 5′ phosphate and 3′ hydroxyl groups. siRNAs mimic miRNA duplexes and enter the RNAi pathway when taken up by RNA-induced silencing complex (RISC). Strand selection occurs to remove the passenger strand and activated RISC silences cognate mRNA. (**B**) Short hairpin RNAs (shRNAs) are generally expressed from RNA polymerase III promoters as a single RNA sequence that folds into a stem loop. As mimics of pre-miRNA, shRNA are recognized and processed by Dicer to form siRNAs that then then enter RISC. (**C**) Artificial pre-miRNAs are imperfectly matched stem loop RNAs that resemble naturally occurring pre-miRNA. As such, they are processed by Dicer into miRNA duplexes and subsequently enter RISC. (**D**) The design of artificial pri-miRNAs (apri-miRNAs) is based on the architecture of naturally occurring pri-miRNA and are recognized and processed by the microprocessor complex, exported from the nucleus, processed by Dicer, and then taken up by RISC.

It is ironic that exploitation of the RNAi pathway preceded its complete elucidation. Following the demonstration by Fire and colleagues that introduction of long double-stranded RNA into the nematode worm caused potent and specific silencing [36], numerous studies used this approach to suppress gene expression. Initial attempts to induce RNAi in mammalian cells however were met with failure. This was because long duplex RNA induces an interferon response that leads to non-specific gene silencing and apoptosis. Elucidation of the pathway revealed that large double-stranded RNAs are processed to 21–23 nucleotide duplexes termed small interfering RNA (siRNA) (Figure 2A) [37]. siRNAs were further shown to have 5′ phosphates, 2′ hydroxyls, and overhangs of two nucleotides at their 3′ ends. Subsequently, chemically synthesized short RNA duplexes with these characteristics were shown to be capable of gene silencing in vitro [38]. When tested in mammalian cells, these siRNAs achieved silencing without inducing the interferon response [39]. This was a seminal study in the field as it broadened the scope of RNAi and paved the way for its use as a therapeutic modality. 

Use of DNA to express RNAi activators was also explored at an early stage. This entailed expression of each individual strand of an siRNA from two expression cassettes [40] or the transcription of short hairpin RNAs (shRNAs) (Figure 2B) [41]. RNA polymerase III promoter-driven shRNA expression proved to be very effective, but given knowledge of RNAi at the time, it was unclear how hairpins achieved gene silencing. It is now clear that shRNAs induce the RNAi pathway by simulating precursor miRNA and siRNAs by mimicking mature miRNA duplexes. This simple concept underlies the basis of reprogramming the RNAi pathway by synthetic or expressed exogenous sequences that mimic various intermediates of the pathway. Later generation exogenous activators of RNAi were designed to resemble miRNAs more closely and are aptly named artificial miRNA (Figure 2C,D).

The discovery that RNAi could indeed be triggered in mammalian cells by synthetic siRNA [39] led to advancing use of the silencing pathway against viral infections. HBV was no exception and there was a concerted effort to achieve siRNA-mediated silencing of the viral genes in vitro and in vivo [42,43,44,45,46]. Initial studies were promising and demonstrated that targeting HBV RNA suppressed replication potently. Because of the arrangement of the HBV genome, with the virus producing overlapping transcripts with a common 3′ end, a single siRNA could simultaneously affect multiple viral factors. 

The designs of synthetic siRNAs were such that the structural features did not deviate significantly from those established in earlier studies [47] and invariably comprised a 19–21 nucleotide duplex with overhangs of two nucleotides at the 3′ ends. Subsequent efforts explored chemical modification as a means of improving siRNA efficacy. Initial reports explored use of 2′-OH modifications (2′-fluoro and 2′-O-methyl), inclusion of deoxyribonucleotides and use of phosphorothioate linkages [48,49]. Combinations of these modifications were introduced into both strands of the siRNA and evaluated in vitro and in mice. Modifications improved in vitro stability of siRNA from a few minutes to hours, and silencing efficacy was not compromised. In vivo, the modified siRNAs outperformed their unmodified counterparts. Formulation of the modified siRNAs into lipoplexes then delivery to transgenic mice stably expressing HBV inhibited viral replication with high efficacy [49]. Moreover, with an initial 3-day daily dose of lipoplexed siRNA, followed up with a single weekly dose, silencing was maintained for up to 6 weeks. More recently, the activity of synthetic siRNA containing novel modified ribonucleotides was explored [50,51,52].

Incorporation of altritol, a six-membered ring in place of ribose, into synthetic anti-HBV siRNA improved silencing activity but at a cost of some hepatoxicity in vivo [50]. In contrast, altritol-modified siRNA exhibited an improved immunostimulatory profile when compared to unmodified siRNA. Incorporation of 2′-O-guanidinopropyl-modified ribonucleotides similarly showed improved stability, silencing, and immunostimulatory profiles [52]. The ease of manufacture of siRNA and compatibility with non-viral delivery vehicles have spurred their rapid adoption and implementation for therapeutic use. This is highlighted by the fact that there are several clinical trials already underway assessing the efficacy of anti-HBV siRNAs (clinicaltrials.gov).

Although chemical modification to siRNAs may increase their half-lives to prolong therapeutic silencing, repeated dosing is required to treat chronic HBV infection effectively. Renewable expression of RNAi activators from DNA cassettes has the potential for more durable silencing. RNA polymerase III-driven transcription, which naturally produces short defined RNA sequences, was initially explored for expression of RNAi activators [41]. Transcription of shRNA sequences (Figure 2B) from RNA polymerase III promoters, first described by Brummelkamp et al. using the H1 promoter [41], proved very popular and remains an important method of gene silencing. The robust U6 promoter has been employed to express anti-HBV shRNA and was shown to silence viral replication with good efficacy in cell culture and in vivo [53]. A caveat of strong shRNA expression is that endogenous miRNA processing may be disrupted to cause severe toxicity in mice [54]. Expression of anti-HBV shRNA from the H1 promoter [55] and the tRNA^Lys^ promoter [56] also silenced viral replication effectively. Characterization of miRNA mechanisms of action fueled research aimed at exploring RNAi activators that more closely resemble these natural gene silencers. Using known miRNA sequences and structures as scaffolds, researchers have developed artificial miRNAs resembling pre-miRNAs and pri-miRNAs (Figure 2C,D) [57,58]. In particular, artificial pri-miRNAs (apri-miRNAs) are very useful because they allow expression from RNA polymerase II promoters, thereby enabling better transcriptional control of RNAi activators [58] and avoidance of toxicity caused by overexpression. Furthermore, as pri-miRNAs often exist as polycistronic sequences, this feature may be adapted to produce multi-targeting miRNAs from single DNA cassettes. This is particularly useful to limit emergence of viral escape mutants [58]. Another advantage of expression cassettes is their compatibility with efficient viral vectors. This was verified by incorporation of anti-HBV shRNA expression cassettes into hepatotropic adenoviral vectors that effectively suppressed HBV replication in transgenic mice [59,60]. This approach was expanded to using lentiviral [61] and adeno-associated viral vectors (AAVs) [62] to deliver HBV-targeting polycistronic apri-miRNA. Using AAVs, inhibition of viral replication was effective for up to 40 weeks in transgenic mice. Considering the importance of long-term silencing for treatment of chronic HBV infection, this is a significant finding.

### 5.4. Significance of Genotype Variability for Advancing RNAi-Based HBV Therapy

HBV is considered a good target for potential treatment on the basis of RNAi [53,59] but sequence variation among genotypes and subgenotypes could influence silencing efficacy. Given the sensitivity of gene silencers to target sequence changes, designing siRNAs, shRNAs, and artificial micro RNAs (amiRNAs) against the conserved sites is essential to ensure activity across multiple genotypes. HBV has been classified phylogenetically into nine genotypes, which are based on intergroup divergence of more than 7.5% across the complete genome (reviewed in [63]). There is a putative 10th genotype, J, isolated from a single individual and predicted to be a genotype C recombinant, but further characterization is required [64]. Genotypes A-D, F, H, and I are classified further into at least 35 subgenotypes on the basis of intergroup nucleotide differences across the complete genome. Genotypes and certain subgenotypes have distinct geographical distributions and differ in their clinical manifestations. For example, subgenotype A1 predominates in Africa, whereas subgenotype A2 is mainly found in Europe. Genotype A is associated with severe liver disease and progression to hepatocellular carcinoma. The HBeAg seroconversion, which is an important intermediate stage in the evolution of chronic hepatitis B (CHB), occurs earlier during genotype A1 infection when compared to genotype A2 and D infection [65]. Genotype C infection is associated with more frequent progression to liver cancer than genotype B infection [66]. Different genotypes also differ in response to antiviral therapy. Genotype A-infected patients respond better than genotype D-infected patients to IFN-α [67,68]. In a recent study, genotype A and D responded poorly to tenofovir but was found to be effective against genotype C and B infection [69,70]. These differences in clinical manifestation and response to therapy may be as a result of subtle viral genomic sequence variation, although host factors may also play a part. Supporting this notion is the fact that nucleotide variability in precore/core (preC/C) sequences of subgenotype A1 and genotype D accounts for differences in expression of HBeAg and efficiency of pregenomic RNA (pgRNA) packaging [71]. Another example is the A1762T/G1764A sequence variation located in the basal core promoter (BCP), which is responsible for controlling pgRNA transcription and is associated with a higher viral load in patients infected with genotype C [72].

In a study aimed at evaluating efficacy of expressed shRNAs against genotypes A-H, the researchers designed 21 shRNAs to target conserved regions [73]. Six of the shRNAs were highly effective and in some cases could tolerate mismatches between the guide and target. One of the effectors, sh10, reduced viral DNA by ≥95%, despite having one mismatch in targets of genotypes A, B, and E and two mismatches to cognates of genotype G and H. This efficacy equaled that of silencers with perfectly complementary targets. In another study, 40 shRNAs were designed to target conserved sequences of genotypes A-I [74]. Four shRNAs were effective against genotypes A-D and I, and inhibited HBV gene expression by up to 90%. However, the inhibitory efficacy of these shRNAs varied significantly against the different genotypes. Employing polycistronic RNAi activators, which produce multiple guides, may be a useful means of overcoming problems of silencing varied sequences with one effector [58]. Overall, it is clearly important that anti-HBV RNAi therapy regimens be tested in the panel of described HBV genotypes to be able to achieve broad efficacy in most chronic carriers of HBV.

## 6. Models of HBV Infection

To advance RNAi-based therapy for HBV infection, use of models that accurately simulate HBV infection and replication is important. Improved understanding of the molecular biology of HBV replication has been beneficial, but suitability and convenience of models that reproduce all stages of HBV infection remain a concern. A factor that has impeded progress was incomplete understanding of the mechanism of HBV entry into hepatocytes. Until fairly recently, cultured cells that could be infected with the virus were limited to primary human hepatocytes (reviewed in [75]) and the HepaRG cell line [76,77]. Description of the NTCP quickly led to generation of HBV-infectable cell lines that had been stably transfected with sequences encoding the receptor [8]. Transfection of liver-derived cells with plasmids that encode reporter constructs that are fused to HBV sequences or greater-than-genome-length replication-competent HBV DNA have been popular and widely used [78,79]. Cells with stably integrated replication-competent HBV DNA have also been used to test antivirals [80,81]. Although convenient, these cell lines do not recapitulate all stages of HBV replication. Differentiation of induced pluripotent stem cells (iPSCs) into HBV-infectable hepatocytes and liver organoids is finding application [82,83].

Preclinical studies in mice have commonly relied on plasmid-derived [48,57,58] or transgenic [62] HBV replication as mice are not natural hosts of hepadnavirus infection. With the hydrodynamic injection mouse model, rapid tail vein injection of a large volume containing replication-competent HBV plasmid results in viral replication in the liver with production of viral antigen markers in the serum [84,85]. This model is useful, but the high-pressure injection may be technically challenging and usually results in transient liver damage. Delivery of replication-competent greater-than-genome-length HBV sequences using AAVs has also been used to replicate HBV in mice [86]. Transgenic mice with integrated DNA comprising greater-than-genome-length HBV sequences replicate the virus in vivo in a model that mimics chronic human infection [86,87]. Although these mice are transgenic, difficulties have been that HBV gene expression is variable and development of antibodies to HBsAg complicates reliability of HBsAg measurements. Ducks and woodchucks can also be used as models of hepatitis infection but are only infected by species-specific hepatitis strains (DHBV and WHV, respectively) [88]. However, genetic and immunological differences of these animals makes inferences from studying these animals to the human condition unpredictable. Asian tree shrews (*Tupaia belangeri*) can be infected by HBV, but these animals have not yet been widely used for evaluation of RNAi-based treatment of HBV infection [89].

Chimpanzees are the only primates susceptible to HBV infection and are an excellent model of chronic HBV infection. However restrictions on use of chimpanzees, high costs, and ethical concerns have limited their use as a preclinical model [90]. Alternatively, other non-human primates may be rendered susceptible to HBV infection. Expression of the human sodium-taurocholate co-transporting polyepeptide (hNTCP) in macaques using a helper-dependent adenoviral (HDAd) vector permitted in vivo infection with HBV and markers of replication were detected for up to 6 weeks after infection [91]. This is a promising HBV infection model, but further optimization is still required to ensure long-term HBV expression.

## 7. Delivery of HBV-Targeting Gene Silencers

An important goal in the translation of RNAi-based therapies for HBV is targeted delivery to the liver. Several delivery strategies have been investigated, which include synthetic non-viral formulations and recombinant viral vectors, which are used for delivery of synthetic and expressed RNAi activators, respectively.

### 7.1. Non-Viral Vectors

Lipid nanoparticles (LNPs) were used in a number of earlier studies to deliver siRNAs (reviewed in [92]). These vectors comprise nucleic acid-binding lipids together with other compounds that assemble to form the LNPs. Hepatocyte-targeting compounds, such as galactopyranoside cholesterol, and PEG to improve stability in blood are also often used in formulations. Uniform small size of the LNPs (<100 nm diameter) is important to avoid sequestration during circulation and facilitate traversing of the sinusoidal fenestrations to reach hepatocytes. Features that promote efficient endosomal escape are also required to augment cytoplasmic siRNA delivery.

In an early study, chemically modified siRNAs targeting HBV were incorporated into stable nucleic acid lipid particles (SNALPs) then administered by intravenous injection to mice replicating HBV [49]. This SNALP-formulated siRNA had a longer half-life in the liver and improved efficacy compared to unformulated siRNAs. Reduced HBV DNA was detected in the serum for up to 6 weeks following weekly dosing. Pegylated nanoparticles were used successfully to target unmodified siRNAs to the liver [93]. Following repeated systemic administration, threefold reductions of markers of HBV replication were observed in HBV transgenic mice over a 28 day period. Hepatotropic lipoplexes containing guanidinopropyl-modified siRNAs targeting *X* also effectively silenced HBV replication [52].

More recently, conjugate-mediated delivery of siRNAs has gained momentum in preclinical and clinical applications. siRNAs conjugated to *N*-acetylgalactosamine (GalNAc/NAG) have become a popular choice for liver-targeted delivery. Conjugation of siRNAs to ligands derived from GalNAc enables uptake by the asialoglycoprotein receptor (ASGPR) on hepatocytes, resulting in liver-specific delivery of siRNAs in vitro and in vivo [94]. A single co-injection of GalNAc-conjugated melittin-like peptide (NAG-MLP) with a cholesterol conjugated HBV-targeting siRNA successfully reduced HBV RNA, DNA, and protein expression in mice [95]. A similar two-vial formulation was used in the well-known clinical trial candidate ARC-520 (Arrowhead Pharmaceuticals), in which two distinct cholesterol-conjugated siRNAs were mixed with NAG-MLP prior to injection. ARC-520 initially showed promising preclinical and clinical results [96,97], but lethal toxicity of the EX1 dynamic polyconjugate (DPC) delivery vehicle, a version of NAG-MLP, in a related safety study in non-human primates led to the discontinuation of the ARC-520 clinical trial (Table 1).

In a recent advancement, GalNAc was modified to produce a triantennary GalNAc ligand that enabled robust and durable gene silencing in the liver following subcutaneous administration to mice [98]. In further refinement of this formulation, 5′ siRNA modification significantly enhanced the stability of triantennary GalNAc–siRNA conjugates in mice and non-human primates [99]. The effect was presumably a result of preventing degradation by cellular exonucleases. Strategic positioning of chemical modifications, such as with 2′-deoxy-2′fluoro and 2′-O-methyl ribose, also improved siRNA potency and duration of silencing in non-human primates [100]. Interestingly, fully modified siRNAs appear to be better suited for conjugate-mediated delivery in vivo, and this is irrespective of the siRNA sequence or type of conjugate [101].

### 7.2. Viral Vectors

Viruses have evolved efficient mechanisms of cell transduction, and this feature has been exploited to make viral vectors that have been used successfully to deliver expressed gene silencers in vivo (reviewed in [103]). Typically, these vectors are replication defective and lack essential viral components that are necessary to reproduce after infecting cells. Transgenes are coupled to viral vector packaging signals, and production of the vector particles in packaging cells is enabled by expressing essential constituents in trans. Recombinant viral vectors typically interact with cell surface molecules to facilitate endocytosis then effect a cascade of events that culminate in transgene delivery and expression.

Recombinant lentiviral vectors (LVs), adenoviral vectors, and adeno-associated viral vectors (AAVs) have all shown good transduction efficiency of the liver, and have therefore been a logical choice for delivery of anti-HBV sequences [59,60,61,62]. However, not all the vectors are ideally suited to delivering HBV-targeting silencing expression cassettes. Adenoviral vectors typically display high innate immunostimulation to result in relatively short-term transgene gene expression. Lentiviral vectors transduce adult hepatocytes in vivo with low efficiency and also carry oncogenic risk owing to their chromosomal integration.

AAVs have emerged as good candidates for delivery of gene-based therapies. Initial concerns about oncogenic potential of AAVs [104] were allayed by subsequent investigation [105]. Several pre-clinical studies against HBV and other diseases have demonstrated the safety and efficacy of AAVs in vivo [62,106]. Moreover, a recently published one-year study of a patient treated with Glybera, an AAV-based gene therapy drug for lipoprotein lipase deficiency, confirmed AAV vector safety and efficacy for human application [107]. AAVs have a non-enveloped icosahedral structure, and properties of the capsid protein from different numbered serotypes determine features of the vectors. For example AAV8 is hepatotropic, while AAV5 efficiently targets cells in the brain [108]. The two commonly used types of recombinant AAVs are single-stranded AAVs (ssAAVs) and self-complementary AAVs (scAAVs). These vectors differ according to their genomic DNA conformations, transgene capacity, and expression kinetics [109]. Ideally, the ssAAV and scAAV are capable of packaging about 4.7 kb and 2.35 kb DNA sequence, respectively. Despite smaller transgene capacity of scAAVs, an important advantage of these vectors is their faster transgene expression. Expression cassettes encoding shRNAs or apri-miRNAs are typically small and can usually be accommodated by scAAVs [62,110,111]. Moreover, the interesting observation that HBV infection enhances AAV transduction of hepatocytes makes these vectors well suited to delivery of anti-HBV RNAi expression cassettes [112].

Use of AAVs to deliver anti-HBV shRNAs or miRNAs has been extensively explored [62,110,113]. Delivery of anti-HBV sequences with scAAV8 achieved inhibition of replication of HBV in transgenic mice that lasted the 10 month duration of the study [62]. This was achieved following administration of a low single dose of 1 × 10^11^ vector genome copies per mouse, which was a 10-fold lower dose than that which was reported in a previous study [114]. AAV-based combinatorial strategies that simultaneously target both HBV and host fibrotic mediators such as transforming growth factor-beta (TGF-β) have been shown to improve efficacy [113]. Using AAVs to deliver antiviral RNAi effectors, together with components of the RNAi machinery such as Argonaute 2 or sense strand inhibitory RNA decoys, avoid endogenous RNAi pathway saturation, reduce toxicity, improve specificity, and enhance efficacy [110,115].

Despite their impressive efficacy and safety profile, rAAVs are not without shortcomings. High prevalence of pre-existing immunity to vectors derived from popular serotypes, such as AAV2 or AAV8, leads to short-lived transgene expression in humans [116,117]. This obstacle is being addressed by AAV capsid re-engineering, de novo rational design of capsids, directed evolution, and in silico vector synthesis [118,119,120,121]. An example of directed evolution entailed use of mutated capsids, generated by DNA shuffling, which were exposed to rounds of selective pressure by neutralizing antibodies [119,122]. This resulted in selection of AAV vectors that evaded immunity and transduced the liver efficiently. Another approach towoards overcoming pre-existing immunity to AAV capsids involved simultaneous administration of transgene-carrying capsids and decoy (empty) capsids [123].

In silico construction of synthetic AAV vectors has been a particularly promising approach to avoiding pre-existing immunity and has been used to generate ancestral genes encoding synthetic AAV capsid variants [120,121]. The method entails several steps. Extant AAV capsids from different serotypes are aligned and phylogenetic methods are then applied to determine ancestral protein sequences. Predicted ancestral DNA sequences are cloned, expressed in transfected mammalian cells, and selected on the basis of sequences encoding capsids that are capable of packaging AAV genomes [121]. Using this approach, Santiago and colleagues compared transduction efficiencies of several ancestral AAV variants and natural AAV serotypes 1–9 in different cell lines. All ancestral AAVs transduced multiple cell lines with more efficiency than AAV2, 4, 5, 8, and 9, although AAV1 and AAV6 showed similar efficiencies [120]. In another study an ancestral vector (Anc80L65) that is antigenically distinct to extant AAV capsids showed enhanced attributes of transduction [121]. Compared to rAAV8, Anc80L65 achieved stronger GFP reporter transgene expression in the muscle, liver, and eye. Moreover, Anc80L65 transduced AAV8 pre-immunized mammals efficiently. These encouraging results based on modification of AAV capsids to improve liver transduction bode well for advancing use of AAVs to delivery HBV gene silencers.

### 7.3. Clinical Trials Evaluating RNAi-Based Treatment for HBV Infection

Use of siRNAs has reached clinical evaluation for numerous diseases, including chronic HBV infection. GalNAc-conjugated siRNAs developed in preclinical studies by Arrowhead Pharmaceuticals [96,97] have been tested in clinical trials for HBV treatment [102] (Table 1). The ARC-520 siRNA was found to be well-tolerated with no serious adverse events in healthy volunteers (NCT01872065, clinicaltrials.gov) [96]. ARC-520 was also well-tolerated and active in patients with CHB in a phase II trial (NCT02065336), resulting in a strong HBsAg reduction in treatment-naïve HBeAg positive patients, but not in HBeAg-negative patients or those previously on long-term nucleoside/nucleotide analogue treatment [97]. This observation was further investigated in chimpanzees and attributed to notable HBsAg expression from integrated copies of the HBV genome, many of which lacked the ARC-520 target site, which was commonly deleted upon integration. In subsequent phase II trials (NCT02604199, NCT02604212), ARC-520 reduced HBsAg expression for at least 85 days in both HBeAg-negative and HBeAg-positive nucleoside/nucleotide analogue-experienced patients [102]. However, the data showed that absolute reductions in HBsAg were moderate, again likely a result of HBsAg expression from integrated HBV DNA.

These studies showed the previously underappreciated contribution of HBsAg expression from integrated copies of the HBV genome, as opposed to cccDNA, and highlighted the need for additional siRNAs capable of targeting all HBV transcripts, regardless of origin. A second-generation siRNA targeting all HBV transcripts, ARC-521, was found to reduce HBsAg and HBV DNA levels in a phase I trial (NCT02797522). However, both ARC-520 and ARC-521 trials were discontinued following the observation of lethal toxicity of the EX1 delivery formulation, a version of NAG-MLP, in a safety study in non-human primates. Subsequently, a GalNac-conjugated siRNA capable of targeting all HBV transcripts (JNJ-3989, formerly ARO-HBV) and administered subcutaneously is under investigation (NCT03365947) and being developed in collaboration with Janssen Pharmaceuticals. Clinical data released thus far indicates that JNJ-3989 is well-tolerated in patients with CHB, with a HBsAg reduction by ≥1 log_10_ IU/mL in all 40 patients and HBsAg < 100 IU/mL in 88% of patients over 24 weeks. Importantly, all measurable viral products were reduced in both HBeAg-positive and -negative patients. JNJ-3989 is currently being investigated in a phase II clinical trials in combination with a nucleoside/nucleotide analogue and JNJ-6379, a capsid assembly modulator (NCT03982186, NCT04129554).

A siRNA delivered intravenously using LNPs (ARB-1467) was also assessed in a phase II clinical trial by Arbutus Biopharma (NCT02631096) (Table 1). The treatment was well-tolerated and both HBsAg and HBcrAg were reduced after multiple doses with a HBsAg reduction of > 1 log in 6 out of 11 patients. In a cohort receiving a biweekly dose, an average HBsAg reduction of 1.4 log_10_ was observed in all 12 patients (https://investor.arbutusbio.com/node/13611/pdf). Despite encouraging results, the development of ARB-1467 has been discontinued. In an ongoing phase I/II study by Vir Biotechnology in collaboration with Alnylam Pharmaceuticals, the VIR-2218 (formerly ALN-HBV02) GalNAc-conjugated siRNA is being assessed in healthy and chronically infected volunteers to determine safety, tolerability, and antiviral efficacy (NCT03672188). Vir Biotechnology has indicated that preliminary results are promising with a durable, dose-dependent reduction in HBsAg, but official data have not yet been published.

## 8. Future of the Field

Over the past 20 years, synthetic siRNAs and expressed activators of the RNAi pathway have proved to be capable of silencing HBV replication in vitro and in vivo. Durability of silencing efficacy achieved by expressed HBV-targeting sequences, such as those that are encoded by apri-miRNA expression cassettes delivered with viral vectors, is a very useful property for treating the chronic disease. However, advancing the technology to enable inexpensive production of viral vectors, avoidance of host immunity, and efficient transduction of hepatocytes need to be tackled successfully before this approach is translated to a clinical setting. Synthetic anti-HBV siRNAs in non-viral formulations are currently at a more advanced stage than HBV-targeting viral vectors, and are being tested in clinical trials. Available clinical data are promising and indicate that anti-HBV siRNAs are safe and well-tolerated. Given the large number of people who are chronically infected with HBV, an important potential advantage of these synthetic drug candidates is that they are more conveniently amenable to scalable and economical production.

Progress with optimizing effectors of gene silencing and identifying good HBV targets has been impressive. Silencers have been directed to sequences that encompass almost the entire HBV genome, but particularly good efficacy does not appear to result from targeting specific viral sequences. Nevertheless, certain viral targets may become more favored for reasons that are not yet completely understood. Improved knowledge about the cellular mechanisms of HBV infection and replication, particularly as they pertain to cccDNA biogenesis, transcription, and degradation, may provide the insights that are necessary to improve selection of viral cognates. HBx has recently been shown to facilitate degradation of the structural maintenance of chromosomes (SMC) 5/6 complex [18,124]. The effect is achieved through action of a cellular ubiquitin ligase, and results in enhanced transcription from cccDNA. It will be interesting to determine whether RNAi activators targeting *HBx* have a particularly suppressive effect on transcription from cccDNA. Inhibition of transcription from cccDNA, especially if it is sustained, will be a significant achievement and go some way to achieving a functional cure for HBV infection. An added benefit of directing RNAi activators to *HBx* is that the sequence is common to all viral transcripts, and inhibition of expression of all viral proteins may be achieved by targeting this gene.

Developing new approaches to economic preparation of vectors, avoidance of host immunity, characterization of pharmacokinetics, exclusion of unintended off-target silencing, and assay of biomarkers of cccDNA in carriers are current priorities. Moreover, available animal models of HBV infection have limitations, and testing candidate drugs in settings that closely simulate natural chronic HBV infection will facilitate drug development. Meeting these goals will provide an impetus. Nevertheless, overall progress in the field indicates that potent, effective silencing of viral transcripts generated from cccDNA in chronic HBV infection is achievable and has therapeutic potential. Results from current and future clinical trials are keenly awaited, and outcomes from these studies will pave the way for achieving the goal of a functional cure for chronic HBV infection.

## Figures and Tables

**Table 1:** Selected clinical trials evaluating gene silencing-based treatment for HBV infection.

siRNA Activator	Delivery	Company	Phase	Identifier (Clinicaltrials.Gov)	End Date	Reference
JNJ-3989 (ARO-HBV)	GalNAc	Arrowhead Pharmaceuticals	I/IIa	NCT03365947 NCT03982186 NCT04129554	September 2020	-
ARC-520	GalNAc	Arrowhead Pharmaceuticals	I, IIb	NCT01872065 NCT02065336 NCT02604199 NCT02604212	Completed/terminated	[96,97,102]
ARC-521	GalNAc	Arrowhead Pharmaceuticals	I	NCT02797522	Terminated	-
VIR-2218 (ALN-HBV02)	GalNAc	Alnylam Pharmaceuticals/ Vir Biotechnology	I/II	NCT03672188 NCT02826018	March 2021	-
ARB-1467	LNP	Arbutus Biopharma	IIa	NCT02631096	Completed	-
